# Supplementary material for: Exact solutions of (1 + 1)-dimensional integro-differential Ito, KP hierarchy, CBS, MCBS and modified KdV-CBS equations
Source: PLoS One. 2023 Mar 30;18(3):e0283569. doi: 10.1371/journal.pone.0283569 (PMC10062672; doi:10.1371/journal.pone.0283569)
Supplement: Attachment — Submitted filename: Response to Reviewers pdf.pdf [file pone.0283569.s001.pdf]

Exact solutions of $(1 + 1)$-dimensional integro-differential Ito, KP Hierarchy, CBS, MCBS and Modified KdV-CBS Equations

We are thankful to the reviewers for their valuable suggestions to improve the article. We have carefully revised the paper according to the reviewers' comments, as we outline in detail below.

Reviewer #1:

Minor comments:

- **Comment 1**: The contents of last five lines in page 3 should be modified.

  Response to comment 1: The contents of last five lines in page 3 were about Sine-Cosine algorithm. Due to some addition in introduction in page 1, the above topic has been described in page 4. Now the Sine-Cosine algorithm is on page 4 and it has been modified.

- **Comment 2**: P4 " We Separate" should be " we Separate". "After computing the values of alpha and beta, put in the main equation, we obtain the solution." should be modified.

  Response to comment 2: In Page 4 " We Separate". and "After computing the values of alpha and beta, put in the main equation, we obtain the solution." have been modified as suggested by the reviewer.

- **Comment 3**: P16. " modified Kdv-CBS equation" should be " modified KdV-CBS equation". "the Similarity transformations" should be "the similarity transformations".

  Response to comment 3: In page 16 "modified Kdv-CBS equation" and in page 17 "the Similarity transformations" have been rectified.

- **Comment 4**: P17. "Now, Using the sine-cosine" should be "Now, using the sine-cosine". "Substituting k = -1 in (7.10), to obtain" should be modified.

Response to comment 4: In page 17 "Now, Using the sine-cosine" has been changed to 'Now, using the sine-cosine". Also 'Substituting k = -1 in (7.10), to obtain" in page 18 has been rectified.

- **Comment 5**: P18. "modified Kdv-CBS equation (7:2)." Should be "modified KdV-CBS equation (7:2)." "integro-differential (Ito)," should be "integro-differential Ito,".

  Response to comment 5: In page 18 "modified Kdv-CBS equation (7:2)." has been changed to "modified KdV-CBS equation (7:2)." and"integro-differential (Ito)," has been changed to "integro-differential Ito,".

- **Major Comments**: English of whole paper should be checked for grammar. In this paper, the Lie point symmetry method is applied to the nonlinear systems. The symmetry reductions related by the nonlocal symmetry are studied in "Commun Nonlinear Sci Numer Simulat 42 (2017) 456; Nonlinear Dyn 86 (2016) 1855; Eur. Phys. J. Plus 131 (2016) 441". The authors can add some discussions in this field.

  Response to Major Comments: As advised by the reviewer, English of whole paper has been checked for grammar. A paragraph in Section-1 (Introduction) has been added on page 1 and page 2, which sheds some light on the relevance of the nonlocal symmetry to the Lie point symmetry of nonlinear PDEs. Appropriate references have been added, and the text is highlighted.

Reviewer #2:

- **One remark**: The authors have stated some important methods which can be used to compute solutions of nonlinear PDEs in introduction. If the authors add the following content to introduction to discuss the latest progress of Dbar-steepest descent method in the section of INTRODUCTION, this paper will be more excellent.

  "Recently, Li, Tian, Yang and Fan have done some interesting work in deriving the solutions of Wadati-Konno-Ichikawa equation and complex short pulse equation with the help of Dbar-steepest descent method. They solved the long-time asymptotic behavior of the solutions of these equations, and proved the soliton resolution conjecture and the asymptotic stability of solutions of these equations. (See: On the soliton resolution and the asymptotic stability of N-soliton solution for the Wadati-Konno-Ichikawa equation with finite density

initial data in space-time solitonic regionsAdv. Math. 409 (2022) 108639; Soliton Resolution for the Wadati–Konno–Ichikawa Equation with Weighted Sobolev Initial DataAnn. Henri Poincare 23 (2022) 2611-2655; Soliton resolution for the complex short pulse equation with weighted Sobolev initial data in space-time solitonic regionsJ. Differ. Equ. 329 (2022) 31–88.)" Authors should carefully discuss these references in their introduction.

Response to one remark:

As advised by the reviewer, above paragraph in Section-1 (Introduction) has been added on page 2. Appropriate references have been added, and the text is highlighted .

We have taken into account all the suggestions of the reviewers and these have enhanced the presentation and results of our paper.

We hope that the article is now in order.

Yours sincerely,

Amina Amin (on behalf of authors)
